# Use of pain neuroscience education, balance evaluation, and exercise for treating chronic pain with kinesiophobia: a narrative review with clinical recommendations

**DOI:** 10.3389/fpubh.2026.1863881

**Published:** 2026-08-18

**Authors:** Nina H. Russin, Daniel S. Peterson, Kevin Pardon, Matthew Perry Martin

**Affiliations:** 1 College of Health Solutions, Arizona State University, Phoenix, AZ, United States; 2 Arizona State University Library, Arizona State University, Phoenix, AZ, United States

**Keywords:** balance, chronic pain, kinesiophobia, movement avoidance, pain neuroscience education, patient engagement

## Abstract

**Background:**

Chronic pain affects approximately 20% of the global population. In primary care settings, chronic pain may account for up to 37.5% of weekly consultations. Most chronic pain is non-specific, complicating diagnosis and treatment. Kinesiophobia and movement avoidance may accompany chronic pain, necessitating a biopsychosocial approach to treatment. Pain neuroscience education and corrective exercise have been proposed as treatment strategies. This review aims to critically examine the relationships among kinesiophobia, movement avoidance, and chronic pain; assess the effectiveness of pain neuroscience education; and present a practical framework to guide diagnosis and treatment within integrated primary care.

**Methods:**

Literature searches in PubMed, PsycInfo, CINAHL, and Google Scholar utilized the search terms chronic pain, kinesiophobia, fear of movement, fear of falling, backache, arthritis, neuroscience education, neuroeducation, balance, and proprioception. Inclusion criteria included English language, peer-reviewed literature published over the past 10 years. Exclusion criteria included studies not published in English and gray literature.

**Results:**

Both kinesiophobia and movement avoidance were significantly correlated with non-specific chronic pain, particularly among older adults. However, the relationship between kinesiophobia and movement avoidance was not direct. Pain neuroscience education combined with strengthening exercises had a positive impact on chronic pain, movement avoidance, and kinesiophobia.

**Discussion:**

Based on their findings, authors collaborated on a treatment strategy for integrated primary care settings, focusing on patient engagement, pain neuroscience education, balance assessment, and corrective exercise. More research is needed to further explain relationships between chronic pain, kinesiophobia, and movement avoidance, and the best strategies for managing these conditions in primary care.

## Introduction

Chronic pain lasting beyond the normal resolution of injury or illness (>3 months) impacts approximately 20% of the global population (1–3). Furthermore, in terms of healthcare utilization, chronic pain may represent up to 37.5% of primary care visits in a given week in developed nations (4). In the United States alone, medical expenses directly related to chronic pain are $530.6 billion annually (5). Chronic pain is particularly prevalent among older adults, affecting up to 34% of US residents aged 65+ (6, 51). Research suggests utilizing a biopsychosocial conceptual approach in the diagnosis and treatment of chronic pain (7), considering not only physical, but environmental and psychological drivers of chronic pain.

Chronic pain is categorized into primary or non-specific pain, and secondary pain, which is related to underlying pathologies such as cancer. Two important conditions are known to contribute to non-specific pain. The first is kinesiophobia, characterized by fear and hypervigilance due to beliefs that movement will result in acute pain (8). A second condition is activity restriction (typically of mobility tasks including activities of daily living) that may ensue from concerns about fall risk (9–11, 55), or as a neuroprotective mechanism to avoid irritating a painful area (such as a backache) (53). While activity restriction may not be directly related to chronic pain, it is related to increases in fall risk due to losses in physical conditioning (12). Further, activity restriction due to fear of falling may exacerbate pain-related sequelae due to reduced physical conditioning and increased risk of fall-related injuries. One in four older adults report falling every year (13), and about 37% of those suffering fall injuries report requiring medical treatment. Following a fall, the patient may adopt a shuffling gait and kyphotic posture, which can contribute to exacerbation of chronic pain and increased risk of subsequent falls (14, 15).

Most chronic pain presenting in primary care is non-specific, (aka primary pain). Among back pain patients, primary pain accounts for approximately 63% of cases globally (16). Primary care providers have access to clinical guidelines for chronic pain management; however, these guidelines often provide limited direction for diagnosing and addressing primary pain mechanisms, such as kinesiophobia and movement avoidance, particularly among older adults with multimorbidity. Pain neuroscience education (also referred to as neuro-education) has been proposed as a valuable addition to non-pharmacologic treatment strategies for chronic pain (11, 17, 30). These strategies also include exercise-based interventions and patient engagement approaches such as motivational interviewing (MI) and cognitive behavioral therapy (CBT). Pain neuroscience education explains the biological and physiological processes underlying the chronic pain experience, including central sensitization and the pain pathway that connects signals traveling from nociceptors in the skin to the brain via lamina II of the dorsal horn in the spinal cord, also known as the “pain gate” [(18, 58)]. This helps patients to understand and accept the idea that pain processing occurs in the brain, and as such, results from a confluence of physical, psychological, and environmental contributors.

The purpose of this review is two-fold. First, the narrative review examines existing therapeutic options for addressing chronic pain accompanied by kinesiophobia, movement avoidance, or both, utilizing pain neuroscience education, balance and strengthening exercises. Following this review, the authors propose a preliminary conceptual framework for evaluating and managing chronic non-specific pain in primary care, intended to translate current evidence into clinical practice and to inform future implementation research.

## Methods

Prior to commencing the narrative review, a concept map was developed, potential keywords were generated and initial searches were conducted in PubMed to identify background articles and assist in the development of the literature search strategy. The initial search was followed by structured database queries conducted in PubMed, PsycINFO, CINAHL, and Google Scholar using the following search string: (kinesiophobia[title/abstract] OR “fear of movement”[title/abstract] OR “fear of falling”[title/abstract]) AND (chronic pain[title/abstract] OR backache[title/abstract] OR “arthritis”[title/abstract]) AND (neuroscience education [title/abstract] OR neuroeducation[title/abstract] OR neuro education [title/abstract] OR “balance”[title/abstract] OR “proprioception”[title/abstract]). The search was translated from the PubMed format above for each database and was reviewed by two authors for accuracy and relevance, to reduce the risk of bias. The searches yielded 179 results after removing duplicates.

Inclusion criteria: articles that addressed the management or treatment of chronic pain accompanied by kinesiophobia were included, along with articles that examined any relationship between movement avoidance and pain, and relationship between kinesiophobia and pain. English language, peer-reviewed literature appearing in scholarly journals between 2016 and 2026, including randomized controlled trials, pilot studies for randomized controlled trials, systematic reviews (with and without meta-analysis), and scoping reviews were also included. The 10 year cutoff was a compromise between being inclusive for relevant research, while acknowledging ongoing changes in neuroscience research and artificial intelligence that could potentially impact neuroeducation research and applications that were the focus of this manuscript. Given the global nature of the subject matter, authors also attempted to include studies reflecting geographically and culturally diverse population.

Exclusion criteria: literature not written in or translated into English language, gray literature, dissertations, conference presentations and abstracts, case studies, and small observational studies with no control group. Since this review focuses on chronic non-specific pain, studies focusing on certain neurological disorders (multiple sclerosis, Parkinson's disease, post-herpetic neuralgia, ankylosing spondylitis, etc.), and other chronic conditions including cancer and stroke survivorship were also excluded, as were studies focused on pre- or post-surgical interventions for chronic pain.

After screening the abstracts of the 179 articles, 15 articles were extracted and included in the narrative review of this manuscript.

### Research questions

The following research questions were developed to guide the narrative review:

1. What evidence exists to support kinesiophobia as a factor influencing pain intensity, pain interference, and pain catastrophizing among older adults living with non-specific chronic pain?
2. What is the association between movement avoidance behaviors, pain intensity, ability to perform activities of daily living, and quality of life among older adults living with non-specific chronic pain?
3. How effective is pain neuroscience education in reducing kinesiophobia, movement avoidance, and subjectively rated pain severity among older adults living with non-specific chronic pain?

## Results

Findings are organized according to the three research questions: (1) associations between kinesiophobia and pain outcomes, (2) relationships between movement avoidance and functional and quality of life outcomes, and (4) the effectiveness of pain neuroscience education. Table 1 lists the research studies extracted for the narrative review (*n* = 15) that follows, including information about study design, participant characteristics, and outcomes. Table 2 lists the countries of origin for extracted studies, the population focus, and key outcome measures.

**Table 1:** Study designs, participant characteristics, and outcomes for included studies.

Authors	Study design	Participants	Outcomes
Bonatesta et al. (31)	Systematic review and meta-analysis of RCTs	*n* = 622	Pain science education plus exercise reduced chronic-non-specific pain symptoms in participants.
Karamanlioğlu et al. (22)	Cross-sectional observational	*n* = 226	Sex, activity-related pain, occupation, education level, disability, and QoL were significant factors in influencing kinesiophobia in persons with chronic low back pain.
Kuzu et al. (26)	RCY	*n* = 60	Aerobic and core stabilization exercises improved ratings of pain and reduced kinesiophobia.
Li et al. (48)	Scientometric analysis of kinesiophobia research 2002–2022	N/A	Authors noticed a trend in chronic pain research toward treatment incorporating physical therapy, cognitive behavioral therapy and virtual reality in treating persons with kinesiophobia.
Malfliet et al. (57)	Multicenter RCT	*n* = 120	Blended-learning pain neuroscience education was able to improve kinesiophobia and illness perception in persons with chronic spinal pain.
Niederstrasser et al. (27)	Cross-sectional	*n* = 172	Pain resilience and male gender but not kinesiophobia were associated with increased physical activity among persons with chronic back pain.
Ortíz-Mallasén et al. (25)	Quasi-experimental, multi-center pilot	*n* = 37, Age: 56.19, Male: 2, Female: 35	Findings from the pilot suggest that therapeutic exercise combined with pain neuroscience education may lead to improvements in QoL, pain catastrophizing, kinesiophobia, pain intensity, and central sensitization.
Pouya et al. (49)	Systematic review and meta-analysis of RCTs	*n* = 6,164	Psychological interventions, including CBT, ACT, graded activity, and education had a small effect on reducing pain intensity and disability short term for persons with low back pain and fear avoidance or catastrophizing.
Romm et al. (30)	Meta-analysis	*n* = 2,440	Therapeutic pain neuroscience education had a statistically significant impact on kinesiophobia among persons with musculoskeletal pain when accounting for dosage and moderator variables.
Saki et al. (24)	Cross-sectional	*n* = 119, Mean age: 67.42, All female sample	Higher pain intensity was strongly associated with kinesiophobia, but there was no association between kinesiophobia and balance.
Shin and Kim (17)	Systematic review and meta-analysis	*n* = 1,038	Pain neuroscience education had positive short terms effects on pain intensity and catastrophizing, and a long-term effect on kinesiophobia.
Tatikola et al. (11)	Systematic review and meta-analysis	N/A	Pain neuroscience education was found to be an effective intervention for reducing perceived pain, pain intensity, pain interference, kinesiophobia, and catastrophizing.
Torres Cruz et al. (23)	Cross sectional	*n* = 80, mean age: 66.98, male: 16, female: 64	Positive correlation between kinesiophobia, pain intensity, and functional disability, with a negative correlation for balance confidence.
Van Bogaert et al. (28)	Secondary data analysis for a multi-center RCT	*n* = 120	Pain neuroscience education combined with exercise therapy decreases baseline kinesiophobia levels on disability, mental health, pain catastrophizing, and hypervigilance over time.
Wagner et al. (50)	Cross-sectional	*n* = 180	The Mini-BESTest for balance evaluation was found to have adequate internal consistency and construct validity for measuring balance in persons with chronic pain. The test had convergent validity with the 10-m walk test and divergent validity with the brief pain inventory, Tampa Scale for Kinesiophobia and Pain Catastrophizing Scale.

**Table 2:** Countries of origin, population focus, and key outcome measures of extracted studies.

Authors	Country of origin	Population focus	Outcome measures
Bonatesta et al. (31)	Spain	Adults aged 40–51 and young adults aged 16–21 with self-reported non-specific chronic spinal pain lasting >3 months.	Pain (VAS), disability (NDI, PDI), kinesiophobia (TSK), catastrophizing (PCS)
Karamanlioğlu et al. (22)	Turkey	Adults with chronic low back pain and kinesiophobia assessed with the Tampa Scale of Kinesiophobia.	Disability (ODI), Kinesiophobia (TSK)
Kuzu et al. (26)	Turkey	Older adults (mean age 70) with chronic non-specific low back pain as diagnosed by a physical medicine specialist.	Walking speed (6 MWT), disability (ODI), depression (BDI), kinesiophobia (TSK)
Li et al. (48)	China	Individuals with chronic pain and kinesiophobia as assessed with the Tampa Scale of Kinesiophobia, participating in studies published between 2002–2022.	Bibliometric analysis
Malfliet et al. (57)	Belgium	Dutch-speaking adults ages 18–65 experiencing non-specific chronic spinal pain at least 3 days/week for a minimum of 3 months.	Disability (PDI), kinesiophobia (TSK)
Niederstrasser et al. (27)	United Kingdom	Adults ages 18+ reporting chronic pain for 3 months or more.	Pain (VAS), Kinesiophobia (TSK), Pain resilience (Pain Resilience Scale), Frailty (FRAIL), recent physical activity (RPAQ)
Ortíz-Mallasén et al. (25)	Spain	Older adults (mean age 56) and 28.89 mean BMI with chronic musculoskeletal pain. 95% of participants were female.	Pain intensity (VAS), pain catastrophizing (PCS), central sensitization (CSI), kinesiophobia (TSK)
Pouya et al. (49)	Canada	Adults ages 18+, with non-specific low back pain, and having a psychological contributor to their pain.	Pain (VAS, NRS), disability (ODI, RMDI)
Romm et al. (30)	United States	Adults with chronic non-specific back pain and kinesiophobia as assessed with the Tampa Scale of Kinesiophobia.	Pain (VAS, NRS), disability (RMDI, PDI, ODI), kinesiophobia (TSK)
Saki et al. (24)	Iran	Women aged 60+ reporting non-specific back pain and kinesiophobia assessed with the Tampa Scale of Kinesiophobia.	Pain intensity (VAS), strength- sit-to-stand, Balance (TUG), kinesiophobia (TSK)
Shin and Kim (17)	Korea	Adults reporting non-specific low back pain for at least 3 months.	Pain intensity (VAS, NRS, BPI), pain catastrophizing (PCS), kinesiophobia (TSK)
Tatikola et al. (11)	India	Adults with self-reported chronic pain.	Pain (VAS, NRS), kinesiophobia (TSK)
Karamanlioğlu et al. (23)	Spain	Older adults with non-specific chronic low back pain and kinesiophobia as assessed with the Tampa Scale of Kinesiophobia.	Pain (NRS), disability (ODI), Activities Specific Confidence Scale, kinesiophobia (TSK)
Van Bogaert et al. (28)	Belgium	Adults diagnosed with chronic non-specific spinal pain assessed with the Tampa Scale of Kinesiophobia.	Pain (NRS), pain disability (PDI), medical outcomes (SF-36), pain vigilance (PVAQ), kinesiophobia (TSK)
Wagner et al. (50)	Sweden	Adults with chronic pain in specialized care assessed with the Brief Pain Inventory, Tampa Scale of Kinesiophobia, and Pain Catastrophizing Scale.	Pain (BPI), kinesiophobia (TSK), pain catastrophizing (PCS), 10-m walk test

### Associations between kinesiophobia and pain outcomes

Multiple studies report a positive and robust association between chronic pain and kinesiophobia, as described in the fear avoidance model (19, 20). The fear-avoidance model describes multidimensional developmental processes involved in the transition from acute to chronic pain, that involves behavioral, psychological, and cognitive aspects of learning (20). The model combines two components: classical (avoidance) and operant (fear). The classical component refers to the neural stimulus acquiring a negative valence over time (20). Based on previous experience of pain elicited by certain movements, the individual predicts similar results (e.g., pain) when performing similar movements, whether or not the movements elicit an actual nociceptive stimulus when performed. The operant component, fear, is an emotional reaction to movement. This reaction can simply promote avoidance, or it can be phobic: an exaggerated and irrational expectancy of pain (20). This irrational fear is known as kinesiophobia.

All of the studies reviewed utilized the Tampa Scale for Kinesiophobia (TSK), a measure initially developed by Miller, Kori and Todd in 1990 to discriminate between “non-obsessive fear of movement and phobia” among subjects with chronic musculoskeletal pain (21). The scale can be used to predict if and when patients' fear of movement may progress to phobia, and has frequently been utilized to assess the relationship between levels of pain and related emotional distrress (11, 22–24, 57). Indeed, higher pain intensity has been associated with higher kinesiophobia in multiple studies, including a cross-sectional study of 199 women aged 60 and over with non-specific low back pain (24), in a multicenter pilot study of primary care patients experiencing chronic musculoskeletal pain (25), and a RCT involving 120 people with non-specific chronic spinal pain (57). Interestingly, two of these studies found no association between kinesiophobia and balance (23, 24), while two others suggest an indirect association (26, 27).

### Effectiveness of pain neuroscience education

Pain neuroscience education (PNE) is a patient education strategy that explains the biological, physiological, and psychological drivers of chronic pain in layperson's terms (18). PNE appears to be most effective in addressing kinesiophobia when it is part of a multimodal chronic pain program (11, 25, 26, 28, 31) which may include other behavioral interventions such as motivational interviewing and cognitive behavioral therapy (29), along with exercises to strengthen muscles and improve functional capacity. A question arises regarding the optimal dosage of pain neuroscience education, given limited resources in primary care (30). There was significant heterogeneity between studies regarding delivery method, the number of sessions and their frequency within the overall rehabilitation program. The studies reviewed make it difficult to draw conclusions about the best “dosage” of pain neuroscience education, with interventions ranging from a single session to multiple weekly sessions delivered over a period of months (11), and one study concluding that the mere “content” of targeted pain neuroscience education had a positive impact on outcomes, including the Tampa Scale for Kinesiophobia, Numeric Pain Rating scale and Visual Analog Scale (30).

A systematic review and meta-analysis covering 13 RCTs (31) and a multicenter pilot study (25) described pain neuroscience education delivered in group format ahead of group exercise therapy, whereas secondary analysis of a RCT conducted at an academic research center (28) employed one-on-one pain neuroscience education and one-on-one exercise therapy. Finally, a systematic review and meta-analysis of nine RCTs involving 1,038 participants reported both individual and group delivery methods. One meta-analysis found that group delivery of pain neuroscience education was more effective than individual sessions for reducing kinesiophobia (30). Authors suggested the role of social learning theory, which posits that learning results from the observation of the behavior of other individuals (32). Study authors suggested that patients in group-based treatment could be positively influenced by observing other group members having less fear of movement (30).

A number of studies mention the association between kinesiophobia and central sensitization: changes in the nervous system resulting in amplification of pain signals coming from the periphery, resulting in allodynia and hyperalgesia (56). An open question is whether multimodal treatments such as graded exposure to movement combined with PNE can induce neuroplastic changes to reduce central sensitization. Authors of a meta-analysis included in this review suggest changes to the “neural matrix” that may account for positive treatment outcomes sustained at the 6-month follow-up (17).

Despite evidence of efficacy for PNE in addressing kinesiophobia among individuals living with chronic non-specific pain, specific clinical frameworks are lacking. Most of the studies reviewed agreed on the purpose of PNE, which is to explain in layperson's terms the neurobiology, neurophysiology, and psychosocial aspects of primary chronic pain (11, 17, 31, 57). However, lack of clarity regarding specific content, delivery methods, frequency and length of sessions (30), makes it difficult to understand the association between PNE and specific outcomes, including pain intensity, pain catastrophizing, and kinesiophobia.

### Activity restriction, functionality, and quality of life

Reductions in proprioception, vision, and hearing related to normal aging processes may result in maladaptive gait alternations, which can significantly impact physical function and health-related quality of life (HRQoL). Walking involves a swing phase and stance phase: normally the stance phase (both feet on the ground) comprises about 60% of the gait cycle with the swing phase (one foot on the ground and the second foot swinging forward) representing 40% (33). Movement avoidance resulting from balance concerns or kinesiophobia can result in longer stance times, increased step width, reduced step length, and increased step errors and variability. Gait modifications may be physiologic or antalgic: a compensatory mechanism to avoid real or perceived pain (33). Over time, these gait alterations lead to further reductions in muscle strength, which also affects sit-to-stand activities. The loss in physical function has two consequences: loss in ability to perform activities of daily living such as dressing, housework, and grocery shopping, and reductions in social activities.

Research studies have found improvements in kinesiophobia via PNE, behavioral and/or exercise interventions mediate HRQoL (34, 35), by improving physical function and mobility. A secondary analysis of a two-arm RCT found that PNE combined with patient-centered goal setting compared to general exercise advice was more effective in fostering intrinsic motivation and behavior change (increased self-efficacy) (35). In a second RCT, PNE plus exercise (intervention group) was compared with standard physiotherapy (control group) for 170 patients with chronic spinal pain (34). At 6-month follow-up, members of the intervention group reported improvements in disability, kinesiophobia, and HRQoL compared to the control group.

The relationship between kinesiophobia, balance, and balance confidence is complex (23, 24, 26, 27). A cross-sectional study of 172 middle-aged participants reporting chronic pain found a significant relationship between chronic pain, pain resilience, and physical activity levels, but insignificant relationships between chronic pain, kinesiophobia and physical activity (27). A second study examining the relationship between kinesiophobia, chronic pain, functional disability and balance confidence in 80 older adults found positive correlations between kinesiophobia, pain intensity, and functional disability, but a negative correlation with balance confidence (23). A third study suggests that the relationship between chronic pain, physical performance, and kinesiophobia found no significant relationship between balance and kinesiophobia (24), and suggested that this association was mediated by reduced lower limb strength in older adults. Finally, a pilot study utilizing aerobic exercise to increase functional capacity and reduce fall risks in older adults with chronic non-specific low back pain found significant improvements in all areas, including kinesiophobia, suggesting that both core stabilization and aerobic exercise were important in both improving physical performance, but also chronic pain and related kinesiophobia (26).

## Discussion

The findings of this review highlight that chronic pain in older adults is shaped by complex interactions among physiological, psychological, neurological, and environmental factors. Importantly, the evidence supports a central role for kinesiophobia and movement avoidance in influencing both pain perception and functional outcomes. Consistent with fear-avoidance models, kinesiophobia appears to play a central role in perpetuating chronic pain through its influence on movement avoidance, physical deconditioning, and functional decline. Accordingly, this discussion presents a preliminary, pragmatic framework for evaluating and managing non-specific chronic pain in integrated primary care that incorporates behavioral screening, functional assessment, pain neuroscience education (PNE), and targeted exercise interventions. Given the time and resource constraints in primary care, the proposed framework emphasizes team-based care and task sharing within integrated behavioral health models while recognizing that prospective validation is needed before routine implementation.

There are numerous assessments to capture balance in older adults, and a full list of these measures is outside the scope of the current review. One commonly used measure is the Short Physical Performance Battery (SPPB), which assesses standing balance, gait speed, and sit-to-stand performance (36). The SPPB offers a brief, functionally relevant assessment with established cutoffs for fall risk and clinically meaningful change (37, 52). However, clinicians should be aware of potential ceiling effects in higher-functioning individuals (38). Although the SBBP is relatively brief, a more streamlined balance assessment as described below, combined with the Timed-Up-and-Go (TUG) test may be easier to implement in a busy primary care practice.

**Figure 1:**
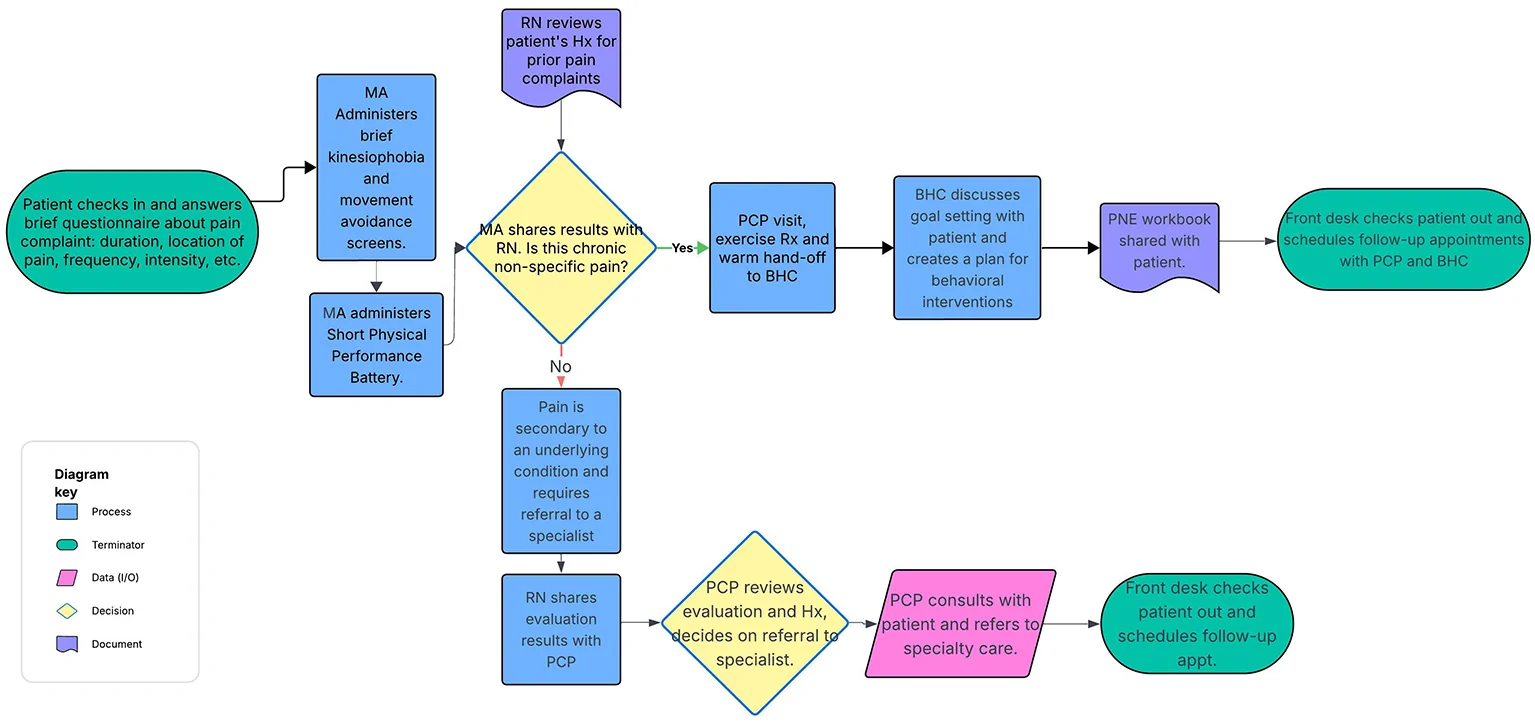
Proposed workflow for chronic non-specific pain, kinesiophobia and movement avoidance.

The primary care framework assumes an average patient visit of 1-h from the time the patient checks in to his/her departure with scheduling to follow-up. Figure 1 illustrates the workflow. Table 3 describes the summarizes the processes illustrated in temporal order.

**Table 3:** Workflow for chronic pain, kinesiophobia, and movement avoidance in primary care.

Activity	Supervisor	Time	Description
Patient check in	Front desk	5 min	Administer PHQ-2, GAD-2, and body outline drawing indicating pain location.
TSK and balance assessment	Medical assistant	10 min	Two questions from the TSK (see discussion), abbreviated SPPB and TUG
Review patient Hx for previous pain complaints	Registered nurse or nurse practitioner	5 min	RN reviews the EHR for previous visits for pain, and determines if the pain is primary (non-specific) or secondary, due to an underlying condition.
Physician consultation	Primary care physician	15 min	PCP determines if the condition can be treated in primary care or requires outside referral. PCP performs a warm handoff to the BHC for patient-centered goal setting.
Behavioral health evaluation	Behavioral health consultant	15 min	BHC reviews chart notes and works with the patient to set meaningful goals for improved function and QoL
Patient check out	Front desk	5 min	Follow-up appointments and referrals if deemed medically necessary.

Based on the findings of this review, a structured, team-based workflow can support the identification and management of kinesiophobia and movement avoidance in primary care settings. Task sharing is essential for maximizing benefits to the patient. At check in, the patient indicates on a body outline drawing the location of the pain. Other intake documents can include PHQ-2 and GAD-2 to screen for depression and anxiety (updated annually). The medical assistant adds an abbreviated version of the Tampa Scale for Kinesiophobia (TSK), and administers shortened versions of the SPPB and timed-up-and-go (TUG) tests. The abbreviated version of TSK (TSK-4) includes questions 3, 6, 7, and 11 from the 17-item TSK (39).

- My body is telling me I have something dangerously wrong.
- My accident has put my body at risk for the rest of my life.
- Pain always means I have injured my body.
- I wouldn't have this much pain if there weren't something potentially dangerous going on in my body. (40).

Scoring is as follows: 1 = strongly disagree 2 = disagree 3 = agree 4 = strongly agree. An answer of three or four on any of these questions suggests some degree of kinesiophobia.

A simplified balance assessment follows. Have the patient stand either in the corner of the exam room or near a sturdy chair or table, so they can catch themselves if they begin to lose balance.

- Feet shoulder width apart;
- Feet together;
- Semi-tandem stance;
- Tandem stance.

Following the balance assessment, the MA will ask the patient to complete the timed-up-and-go (TUG) test, which evaluates leg strength, stability, and gait speed. The patient is seated in a chair (preferably with a straight back and no armrests). The MA asks the patient to cross their arms over their chest, and stand without using their hands to assist. Once standing, the patient walks 3 m (about 10 feet) at a comfortable pace. The patient should be able to stand up from the chair unassisted, and complete the walk in 3.5 s (excellent) to 6.5 s (acceptable). Note if the patient must use their hands to rise up to a standing position, and if the patient uses a mobility aid for walking.

Prior to the PCP encounter, the RN can review the patient's medical history in the electronic health record, including prior pain complaints, duration of symptoms (with pain persisting longer than 3 months suggesting chronicity), and whether the pain may be secondary to an underlying pathological condition (e.g., cancer, rheumatoid arthritis, multiple sclerosis). Although this review focuses on non-pharmacological management strategies, these interventions can complement rather than replace appropriate pharmacological management when clinically indicated. During the primary care evaluation, clinicians can review the patient's current medication regimen, assess effectiveness and adverse effects, and coordinate non-pharmacological interventions with existing pharmacological treatment plans to support comprehensive chronic pain management.

It is important for the PCP to hear the patient's description of the pain complaint in his or her own words. In addition to diagnosing the pain as primary or secondary, it is essential to determine the impact of this pain, kinesiophobia and/or movement avoidance on functionality and quality of life. While certain corrective exercise can be prescribed in primary care, some patients may need referral to physiotherapy, occupational therapy, or both. In the United States, Medicare covers the majority of expenses for medically necessary services of this type (41, 42). Having reviewed the patient's history, impact of the pain on both physical function and mental health, the physician determines to what extent the complaint can be treated in primary care. Ideally, the patient can be seen on the same day by the BHC, to co-create a plan for engagement.

Patient-centered goal setting is also a critical component of chronic pain management, particularly in the context of kinesiophobia and movement avoidance (9). Given that complete pain resolution is often not achievable in cases of non-specific chronic pain, treatment can prioritize improvements in functional outcomes, including activities of daily living (ADLs), mobility, and overall quality of life. Collaborative goal setting between the patient and care team allows for the identification of meaningful, individualized targets that reflect the patient's values, preferences, and daily challenges (43). This approach is especially important in older adults, where maintaining independence and social participation are often primary concerns (44). Incorporating brief behavioral assessments using validated tools (e.g., PHQ-2, GAD-2, and the Tampa Scale for Kinesiophobia) can help identify psychological and behavioral factors influencing pain and inform tailored goal development.

Structured goal-setting frameworks, such as SMART goals (Specific, Measurable, Achievable, Relevant, and Time-bound), can support behavior change by translating broad treatment objectives into actionable steps. Progress toward these goals may be quantified using approaches such as Goal Attainment Scaling (GAS), which enables clinicians and patients to define expected outcomes and track incremental improvements in function and behavior over time (45). When combined with pain neuroscience education and behavioral strategies such as motivational interviewing, goal setting can enhance self-efficacy and reduce fear-avoidance behaviors by encouraging gradual re-engagement in movement and activity (9, 46). Ongoing monitoring through repeated assessments and follow-up visits allows care teams to adjust treatment plans, reinforce progress, and support sustained engagement in self-management strategies (47). Importantly, aligning goals with functional outcomes rather than pain elimination reinforces adaptive coping and supports durable improvements in physical function and health-related quality of life.

The integrated team approach may be challenging for primary care practices lacking behavioral health consultants, necessitating outside referrals. Even if all providers are located within a single medical home, scheduling same-day appointments for all recommended services can be a challenge. Streamlining referral pathways to minimize inconvenience to patients is a priority. This may include checking for insurance coverage and scheduling the referral before the patient leaves the clinic. It is also important to consider methods for communicating with external providers, to ensure that patients are attending follow-up appointments. Finding space within the clinic for exercise training may require some creative thinking. Consider utilizing any spaces which are well lit, and are free of tripping hazards (rugs, steps, furniture). If prescribing home exercises (see below), make sure that the patient is able to obtain any special equipment needed to perform the exercises (such as a balance ball as described below). In some cases, providers may recommend on adjustments to the plan, so patients can utilize what is readily available in the home. For example, if a patient cannot afford an exercise ball, bridging exercises done on a firm mattress or a mat on the floor can have similar benefits.

### Stretching and strengthening exercises

Home exercises prescribed by the PCP can extend the benefits of work done in the clinic, by giving patients the opportunity for more practice. Using an exercise “prescription pad” may increase patient engagement in and long-term adherence with exercise recommendations. By keeping equipment to a minimum, patients can easily do these exercises at home, which eliminates the need for a workout facility. Appendix A includes some basic stretching and strengthening exercises, including the following:

- Hip stretch;
- Hamstring stretch;
- Thoracic stretch;
- Basic bridging;
- Ball wall squat;
- Seated arm extension and twisting exercise on the ball;
- Sit to stand.

The only equipment required for these exercises is a Swiss ball (also called a stability or balance ball), which is relatively inexpensive and widely available online. Swiss balls come in different sizes depending on the person's height. Persons who have not exercised on a Swiss ball before may find it easier to start out by positioning the ball against a wall to add some stability. The strengthening exercises can be performed in 2–3 sets of 8–10 repeats, using slow and controlled movements. Jerky, ballistic movements can increase the risk of injury.

### Long-term considerations

Providers may wish to consider that while chronic pain with movement avoidance and/or kinesiophobia may continue throughout the patient's lifetime, methods of addressing these conditions may need to change due normal aging processes as well as co-morbid diseases such as arthritis, cardiovascular disease (reduced aerobic capacity), diabetes (which may include diabetic neuropathy), overweight/obesity (which can change an individual's center of gravity), and chronic kidney disease (which may necessitate dialysis). Follow-up visits may provide an opportunity to discuss the effects of these conditions, as well as worsening stress, anxiety, or depression, on pain management and functional goals. Ongoing engagement of patients, family members, and caregivers may support long-term adherence to individualized management strategies. In some cases, educating these individuals about lifestyle change recommendations may enhance patient engagement, and sustained improvements in quality of life.

### Limitations

The research around chronic pain, kinesiophobia, and movement avoidance has expanded in recent times, but there are still considerable gaps, and importantly, lack of a specific framework for PNE, which makes comparisons between studies difficult. Because pain is subjectively experienced, patient ratings of pain intensity may be affected by external factors including perceived social support (and social isolation), the therapeutic relationship with clinicians, and pain interference with activities of daily living. This review is limited to studies published in the English language published in peer-reviewed journals. It does not include unpublished literature (e.g., dissertations), or conference presentations and other types of gray literature.

As a narrative review, several methodological considerations can be noted in comparison to other review types such as a systematic review. First, study identification was targeted to capture key thematic concepts rather than an exhaustive review of all available evidence. While two authors were engaged with the creation of the search strategy and selection process to minimize the risk of bias, there still remains an inherent potential risk of selection and publication bias. Second, given the broad scope and heterogeneity of included study designs, a formal risk of bias tool was not conducted in favor of a descriptive appraisal of included studies. This approach ensure that qualitative data related to anxiety, stress, and depression that frequently co-occur with chronic pain, and that may not be measured using quantitative screening tools were retained. Ultimately, the synthesized conclusions can be viewed as a broad conceptual overview of the current literature rather than a systematic, exhaustive evaluation of evidence strength.

## Conclusion

Chronic non-specific musculoskeletal pain, also known as primary pain, is a significant burden of disease, affecting approximately 20% of the global population. Both kinesiophobia and movement avoidance due to fear of pain and further injury appear to contribute to greater pain intensity and reductions in quality of life. Because primary care is frequently the initial point of contact for individuals with chronic non-specific pain (54), it provides an important opportunity to identify and address the behavioral, functional, and physical contributors to chronic pain within an integrated care setting. Based on the literature reviewed, the authors propose a preliminary, pragmatic framework for evaluating and managing chronic non-specific pain in integrated primary care that incorporates behavioral health, pain neuroscience education, balance assessment, and targeted exercise. The framework is intended to facilitate translation of current evidence into clinical practice and to inform future implementation research. Prospective studies are needed to evaluate its feasibility, effectiveness, and applicability across diverse patient populations and primary care settings.
