# Supplementary material for: Use of pain neuroscience education, balance evaluation, and exercise for treating chronic pain with kinesiophobia: a narrative review with clinical recommendations
Source: Front Public Health. 2026 Aug 18;14:1863881. doi: 10.3389/fpubh.2026.1863881 (PMC13531184; doi:10.3389/fpubh.2026.1863881)
Supplement: Supplementary file 1 [file Data_Sheet_1.pdf]

Exercises

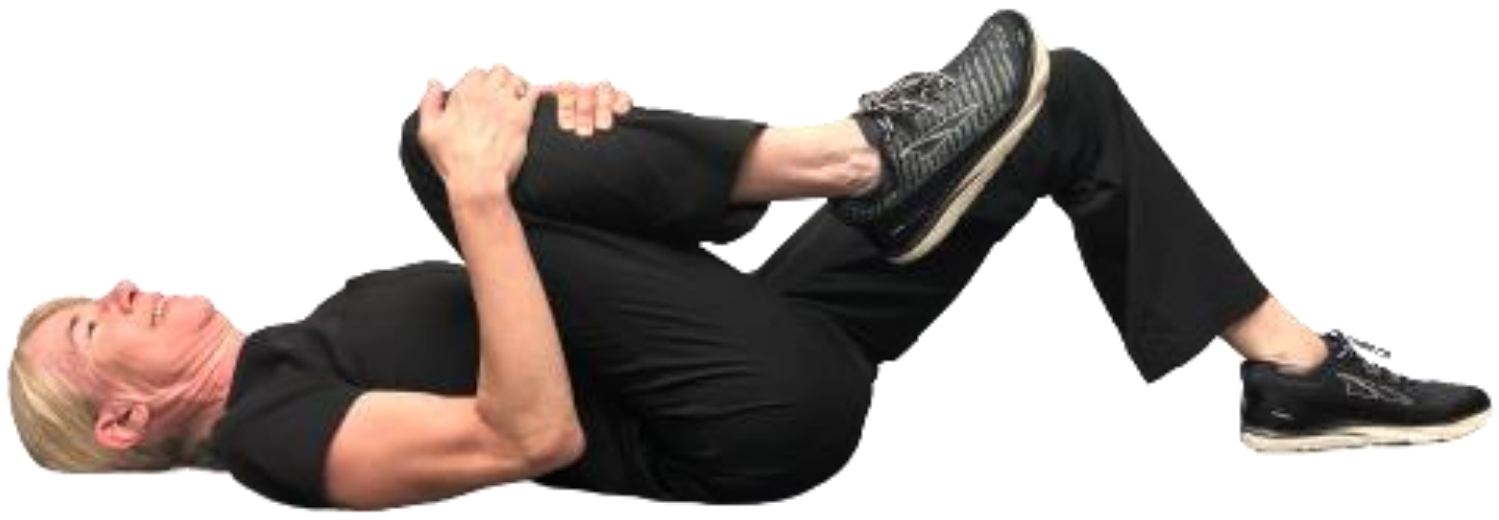

Hip stretch: Begin in a supine position with your feet on the floor or mattress and knees slightly bent. Pull the right knee up towards the shoulders, then gently push the knee towards the left shoulder. Hold the stretch for 15-20 seconds, and repeat on the other side.

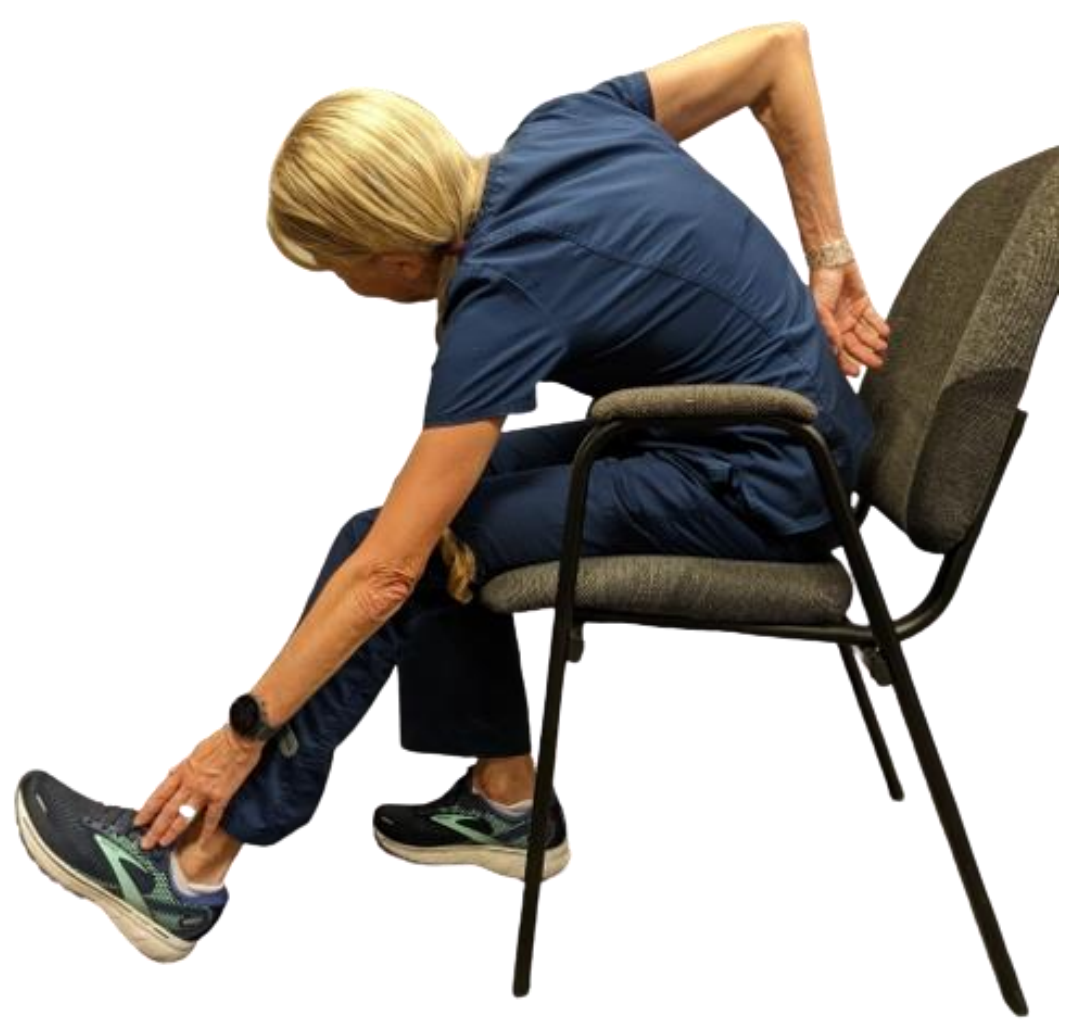

Seated hamstring stretch: Begin by sitting in a chair with a firm cushion and both feet on the floor in front of you. Straighten the left leg slightly. Lean forward and gradually move your left hand down towards the feet. You should feel a comfortable stretch through the left hamstring and lower back. Hold for 15-20 seconds and repeat on the other side.

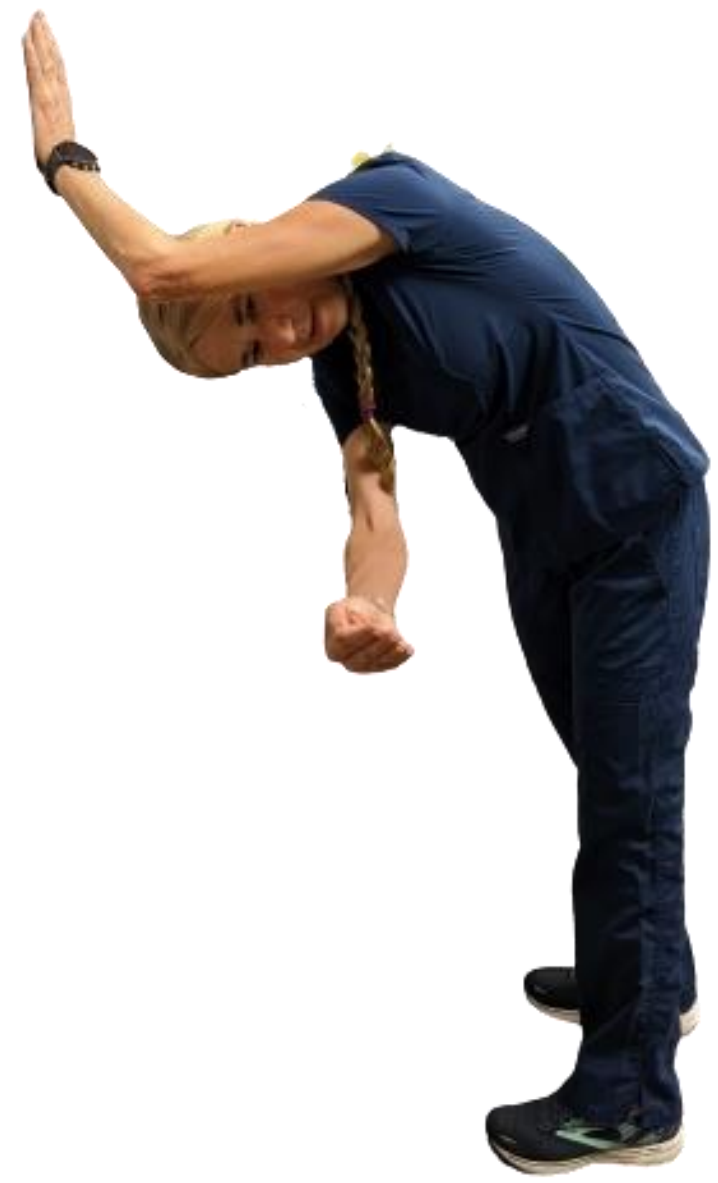

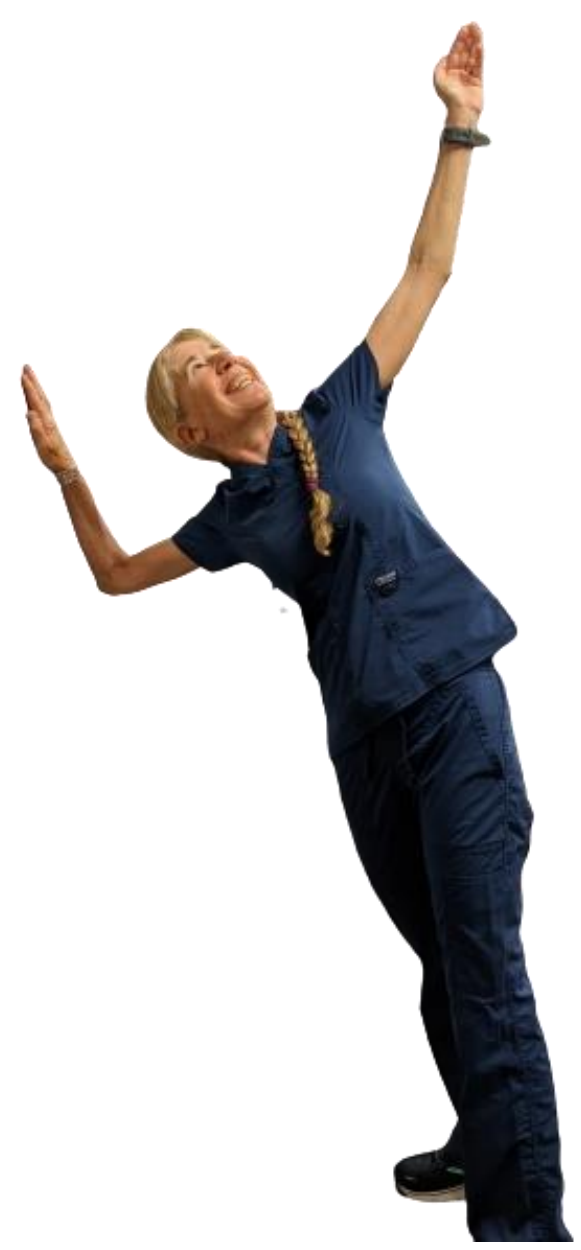

Thoracic rotation: Begin facing the wall with arms bent and hands on the wall. You should be slightly leaning towards the wall. Twist to the left, while extending the left arm up and towards your back. You should feel a nice stretch through your upper chest. Be careful not to arch your

back. Lower the left hand back to the wall and repeat on the right side. Hold each position for 15-20 seconds.

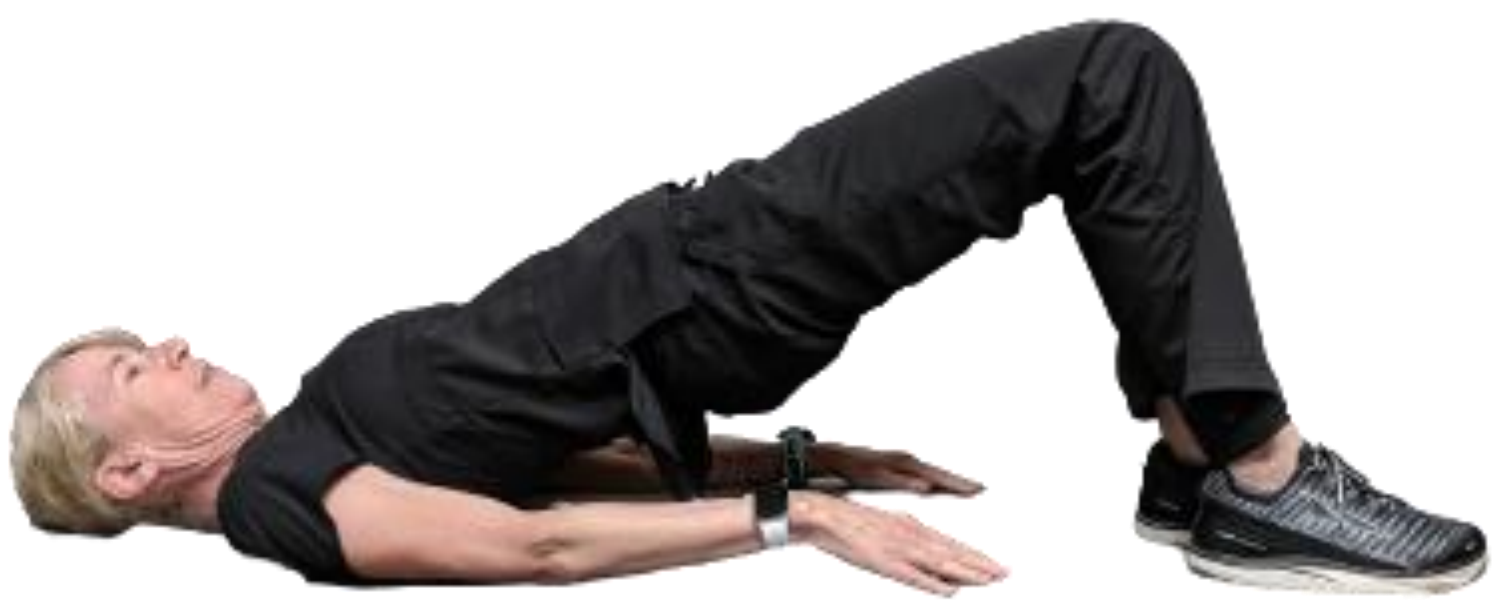

Basic bridging: Begin in a supine position on the floor or a firm mattress. Hips and feet should be on the floor, with the feet shoulder width apart. Gradually raise the hips up so that the area from the shoulders to the hips is a straight line. Be careful not to overextend the hips and arch the back. Slowly lower the hips back down to the floor. Repeat 8-10 times, focusing on slow and controlled movements. To make this exercise more challenging, start with the feet and knees together.

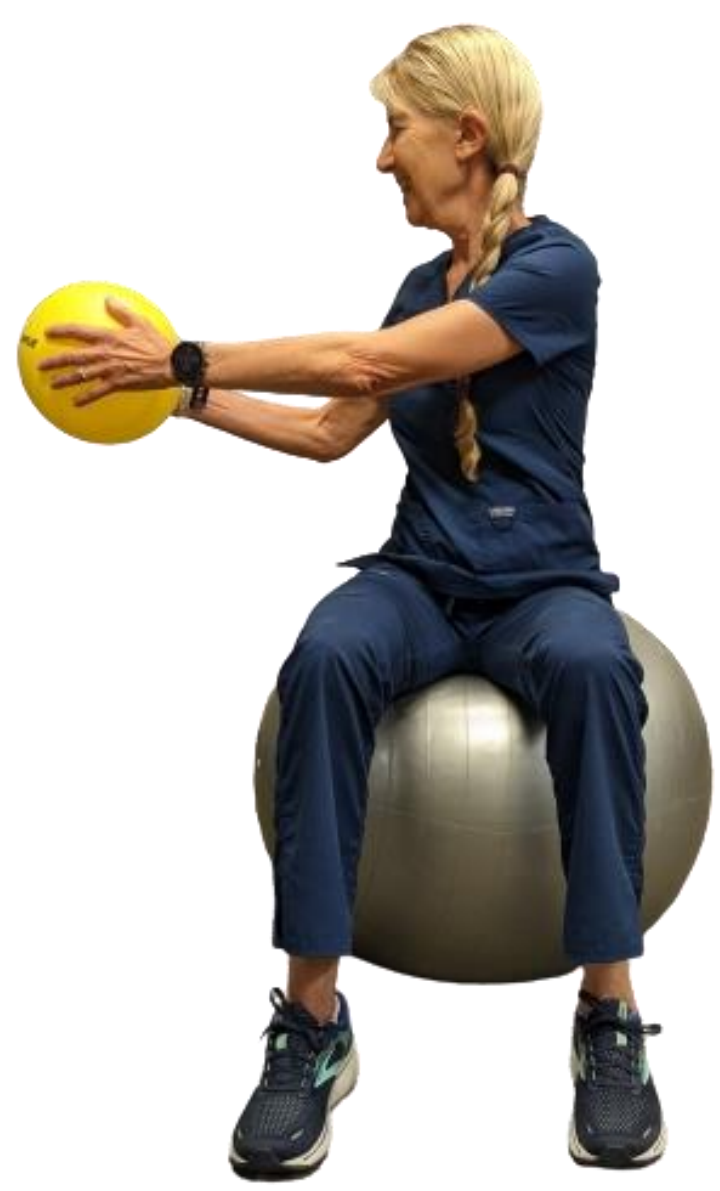

Seated ball rotation: Begin seated on the Swiss ball. If you are not used to sitting on a balance ball, prop it against a wall to increase stability. Hold a small-medium size rubber ball straight in front of you, and slowly rotate to the left and right, focusing on slow and controlled movements. To make this exercise more challenging, move the feet together (see below).

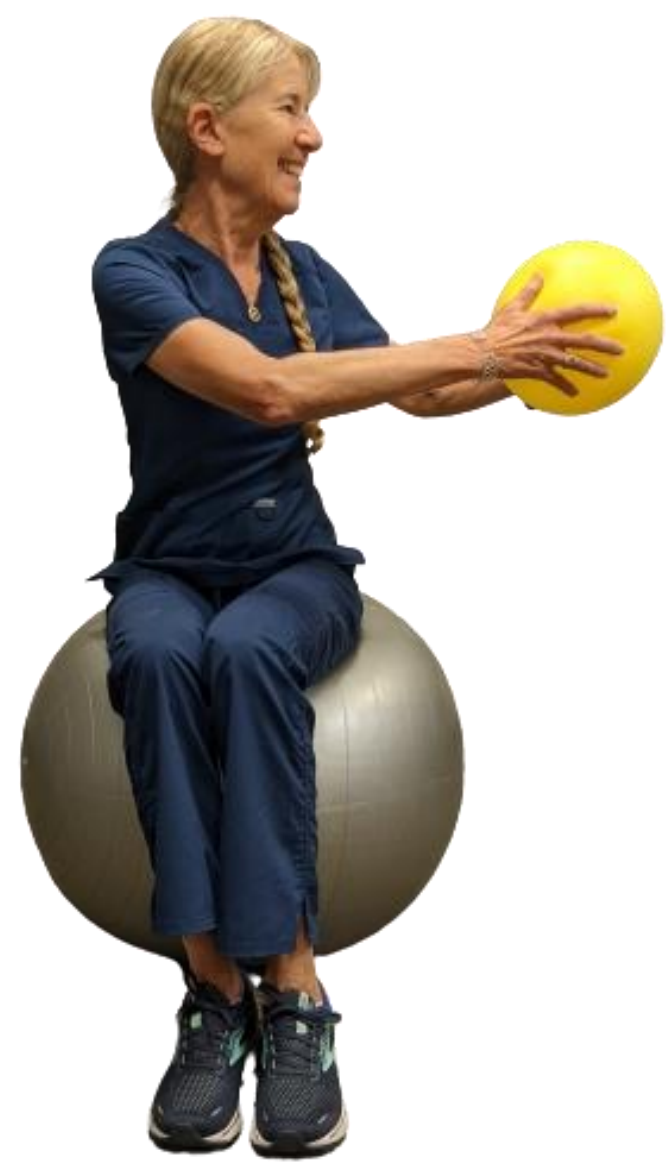

.

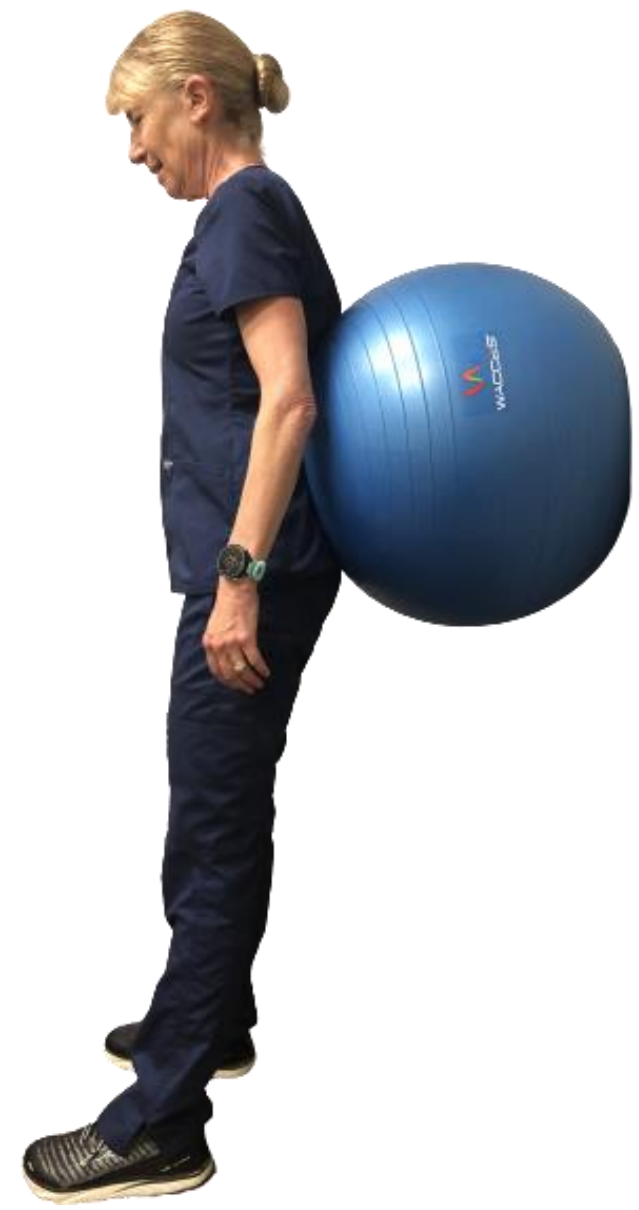

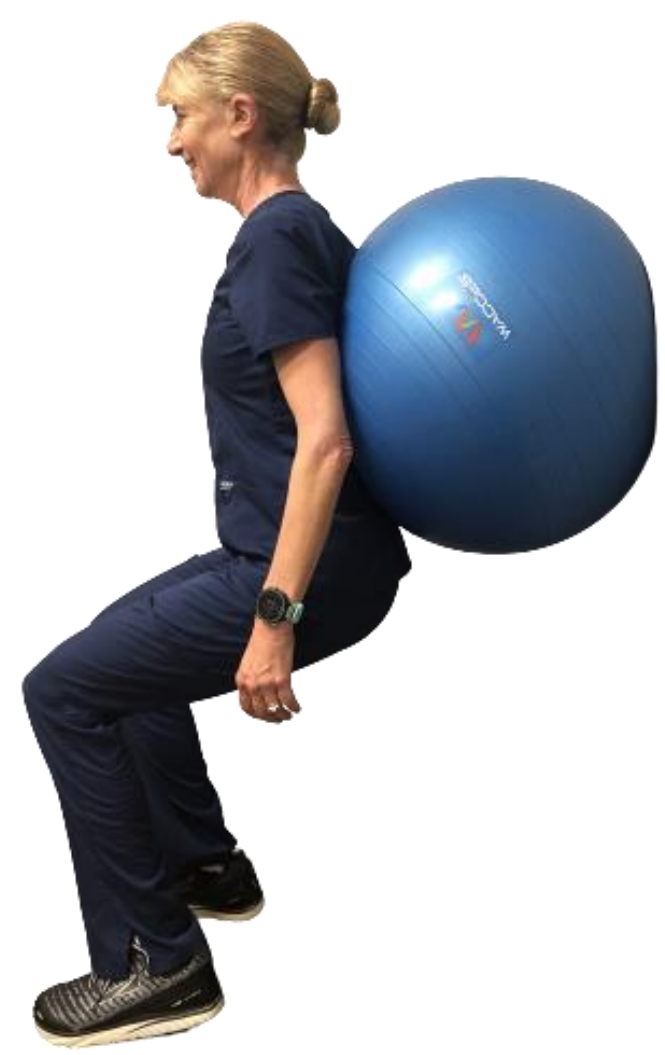

Ball wall squat: Begin at a standing position with the Swiss ball positioned between your back and the wall. Your legs should be straight with feet slightly out in front. Squat down keeping the ball between your back and the wall. Be careful not to extend the knees forward of the feet.

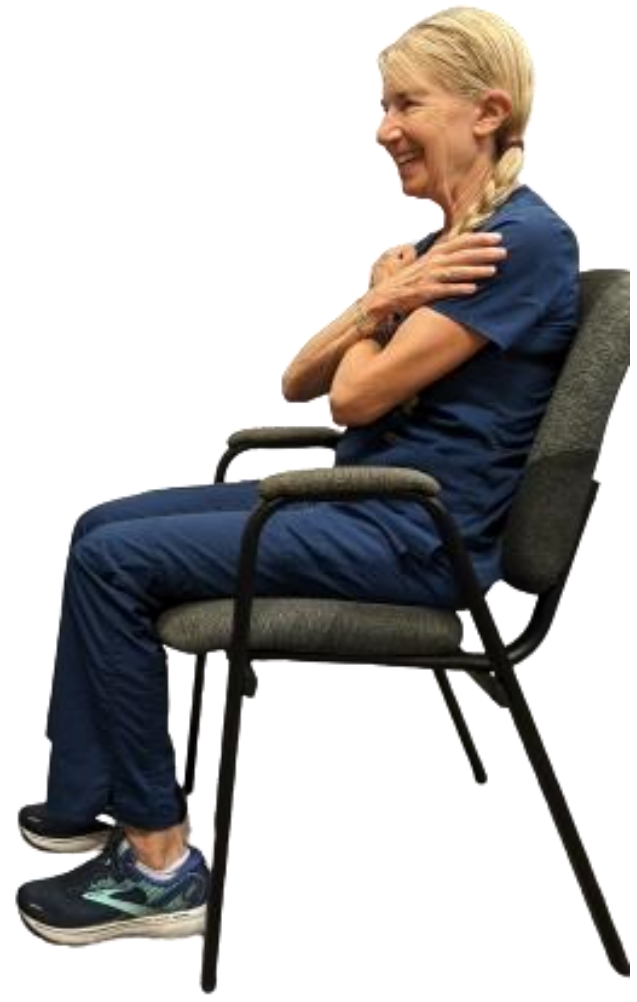

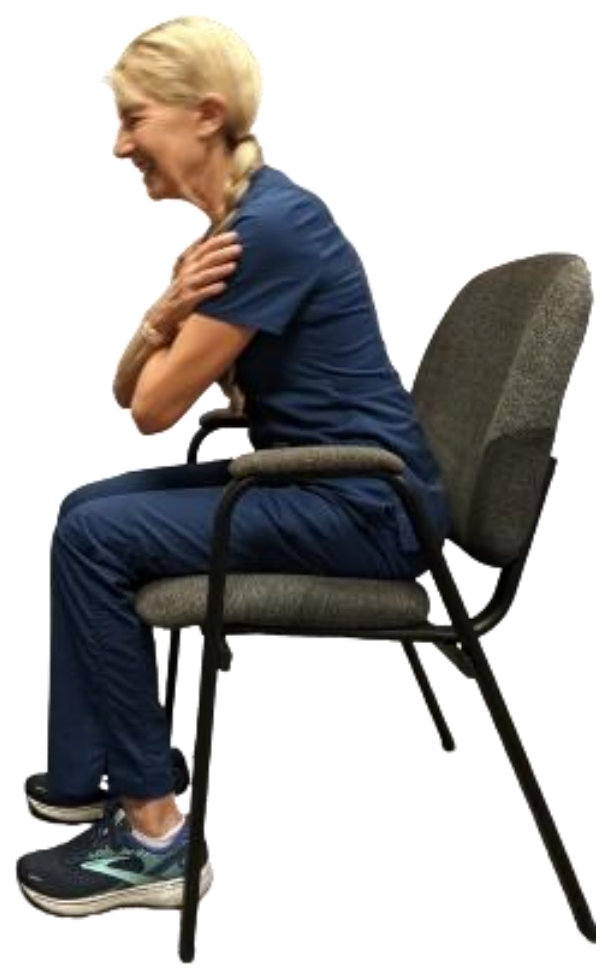

Sit to Stand: Begin seated in a chair with a firm seat cushion and firm back. Lean forward slightly and cross the arms across the chest. Slowly rise from the chair to a standing position, and then slowly lower yourself back into a seated position. Use slow and controlled movements, and avoid using your hands as support. Aim for 8 repeats.
